# Development and Verification of an Immune-Related Gene Pairs Prognostic Signature in Hepatocellular Carcinoma

**DOI:** 10.3389/fmolb.2021.715728

**Published:** 2021-10-01

**Authors:** Xiaofei Feng, Shanshan Mu, Yao Ma, Wenji Wang

**Affiliations:** ^1^ Department of Orthopedics, Lanzhou University First Affiliated Hospital, Lanzhou, China; ^2^ Pediatric Rheumatism Immunology Department, Lanzhou University Second Hospital, Lanzhou, China; ^3^ Clinical Laboratory Center, Gansu Provincial Maternity and Child-care Hospital, Lanzhou, China

**Keywords:** hepatocellular carcinoma, immune-related gene pairs, prognosis, tumor immune environment, targeting therapy

## Abstract

With the increasing prevalence of Hepatocellular carcinoma (HCC) and the poor prognosis of immunotherapy, reliable immune-related gene pairs (IRGPs) prognostic signature is required for personalized management and treatment of patients. Gene expression profiles and clinical information of HCC patients were obtained from the TCGA and ICGC databases. The IRGPs are constructed using immune-related genes (IRGs) with large variations. The least absolute shrinkage and selection operator (LASSO) regression analysis was used to construct IRGPs signature. The IRGPs signature was verified through the ICGC cohort. 1,309 IRGPs were constructed from 90 IRGs with high variability. We obtained 50 IRGPs that were significantly connected to the prognosis and constructed a signature that included 17 IRGPs. In the TCGA and ICGC cohorts, patients were divided into high and low-risk patients by the IRGPs signature. The overall survival time of low-risk patients is longer than that of high-risk patients. After adjustment for clinical and pathological factors, multivariate analysis showed that the IRGPs signature is an independent prognostic factor. The Receiver operating characteristic (ROC) curve confirmed the accuracy of the signature. Besides, gene set enrichment analysis (GSEA) revealed that the signature is related to immune biological processes, and the immune microenvironment status is distinct in different risk patients. The proposed IRGPs signature can effectively assess the overall survival of HCC, and provide the relationship between the signature and the reactivity of immune checkpoint therapy and the sensitivity of targeted drugs, thereby providing new ideas for the diagnosis and treatment of the disease.

## Introduction

Hepatocellular carcinoma (HCC) is one of the most common malignant tumors and the leading cause of cancer-related deaths, and its morbidity and mortality are increasing (Forner et al., 2018; Villanueva, 2019). According to the latest cancer statistics in 2019, approximately 42,030 people in the United States are diagnosed with liver cancer each year, and 31,780 people die from the disease (Siegel et al., 2019). Although breakthroughs in the diagnosis and treatment of HCC have been made in recent years, the prognostic outcome of patients remains poor (Yang et al., 2019). The current clinical application of immunotherapy in HCC has benefited some patients, but this approach has not effectively improved the prognosis, and the long-term survival rate is still very poor (Dong et al., 2020). Due to the complexity and heterogeneity of HCC, individualized decision-making plans are required in diagnosis and treatment. Therefore, it is necessary to identify the novel prognostic signature of HCC and use it to guide clinical treatment as an effective way to improve the prognosis of patients.

Understanding the characteristics of tumor immune cell infiltration can improve the responsiveness of immunotherapy and is of great help in understanding the mechanism of cancer occurrence (Ino et al., 2013; Binnewies et al., 2018). The landscape of immune cells in HCC mapped by single-cell sequencing and other evidence indicate that the tumor immune microenvironment plays an indispensable role in the progression of HCC (Nishida and Kudo, 2017; Zheng et al., 2017; Kurebayashi et al., 2018). Different immune cells infiltrating into HCC have different prognostic effects (Prieto et al., 2015; Cariani and Missale, 2019). It has been observed clinically that the increase of PD1^+^CD8^+^ T cells in HCC is associated with poor clinical outcomes (Chew et al., 2017). Although numerous studies have found the importance of immunology in HCC, its molecular mechanism is still unclear. Current studies have shown that tumor immune-related markers have commendable effects on the diagnosis and treatment of cancer (Peng et al., 2019; Hong et al., 2020). There have been many studies on the prognostic value of identifying key genes to build models to predict the prognosis of HCC patients (Li et al., 2017a; Kaur et al., 2019; Liu et al., 2020; Ouyang et al., 2020). However, there is no in-depth survey on the clinical relevance and prognostic significance of IRGPs in HCC.

In this study, we used the HCC gene expression datasets of the TCGA and ICGC databases to develop individualized prognostic signature based on IRGPs. Then, we evaluated its ability to predict prognosis in HCC patients and its responsiveness to immune checkpoint therapy and targeted therapy.

## Materials and Methods

### Data Sources

The HCC level 3 RNA-seq data and clinical information were downloaded from the TCGA database, including normal tissues (*n* = 50) and tumor tissues (*n* = 374). 365 cases of tumor patients have survival time and survival status, of which 247 cases contain clinical information (age, gender, histologic grade, TNM stage, vascular invasion, and alpha fetoprotein). The RNA-seq data and clinical information of HCC were downloaded from the ICGC database, including normal tissues (*n* = 202) and tumor tissues (*n* = 243). 232 tumor patients have survival time, survival status, and clinical information (age, gender, and TNM stage). The list of IRGs was downloaded from the IMMPORT (Bhattacharya et al., 2018). Both the TCGA and ICGC data are publicly available. Therefore, this research does not require the approval of the local ethics committee.

### Construction of an Individualized Prognostic Signature Based on IRGPs

In the TCGA and ICGC cohorts, the limma package (Ritchie et al., 2015) was used to identify the IRGs that are different between cancer tissues and normal tissues. The filtering criteria were | logFC | > 1, FDR < 0.01. We identified the common differential IRGs in the two cohorts. The protein-protein interaction network was used to demonstrate differential genes (Udhaya Kumar et al., 2021), and the Cytoscape software (v3.8.2) was used for visualization. Genes with the median absolute deviation greater than 1 in tumor samples whose expression levels of these common differential IRGs were considered as candidate genes. The expression levels of these candidate genes were compared in pairs to generate a score for each IRGP. If IRG 1 is less than IRG 2, the IRGP score is 1, otherwise, the IRGP score is 0 (Li et al., 2017b). In the TCGA cohort, univariate cox was used to select prognostic IRGPs to assess the association between each IRGP and the overall survival rate of the patient. Prognostic IRGPs with a *p* value of less than 0.001 were candidates for establishing an IRGPs signature. From these IRGPs, the R language was used to perform Lasso Cox proportional hazard regression to construct the risk score, and finally 17 IRGPs were used to define the IRGPs signature. To classify patients into high- or low-risk patients, the optimal IRGPs signature cut-off value was determined by the ROC curve.

### Verification of IRGPs Signature

In the TCGA and ICGC cohorts, the survival package and survminer package (https://CRAN.R-project.org/package = survminer) were used to establish the survival curve of the high and low-risk patients through the Kaplan-Meier diagram, and the Log-rank test was used to analyze the difference in survival curves. Cox proportional hazards analysis was used for univariate and multivariate analysis. The survivalROC package (https://CRAN.R-project.org/package = survivalROC) was used for ROC curve analysis to evaluate the predictive ability of the IRGPs signature.

### Functional Annotation and Immune Cell Infiltration

To understand the underlying molecular mechanism of the IRGPs signature, we divided the TCGA cohort patients into high- and low-risk patients so that the software GSEA 4.1.0 (http://www.gsea-msigdb.org) can be used for gene ontology (GO) and Kyoto Encyclopedia of Genes and Genomes (KEGG) annotations. When *p* < 0.05 and FDR < 0.25, the enriched gene set is considered to be statistically significant. The gsva package (Hänzelmann et al., 2013) was used to quantify immune cell infiltrations, and the correlation between high- and low-risk patients and 16 immune cell infiltrations and 13 immune-related pathways was evaluated.

### Explore the Relationship Between the IRGPs Signature and the Reactivity of Immune Checkpoint Therapy and the Sensitivity of Targeted Drugs

Immune checkpoint blockers of immunotherapy have obvious effects in the treatment of many human cancers. Therefore, we explored the expression and correlation between high and low-risk patients and the current common immune checkpoints. In addition, small molecule tyrosine kinase inhibitors, as a class of molecularly targeted drugs, have become one of the mainstream trends in current anti-liver cancer research. The pRRophetic package is used to analyze the sensitivity of tyrosine kinase inhibitors in high- and low-risk patients (Geeleher et al., 2014).

## Results

### Construction and Definition of the IRGPs Signature

**FIGURE 1:**
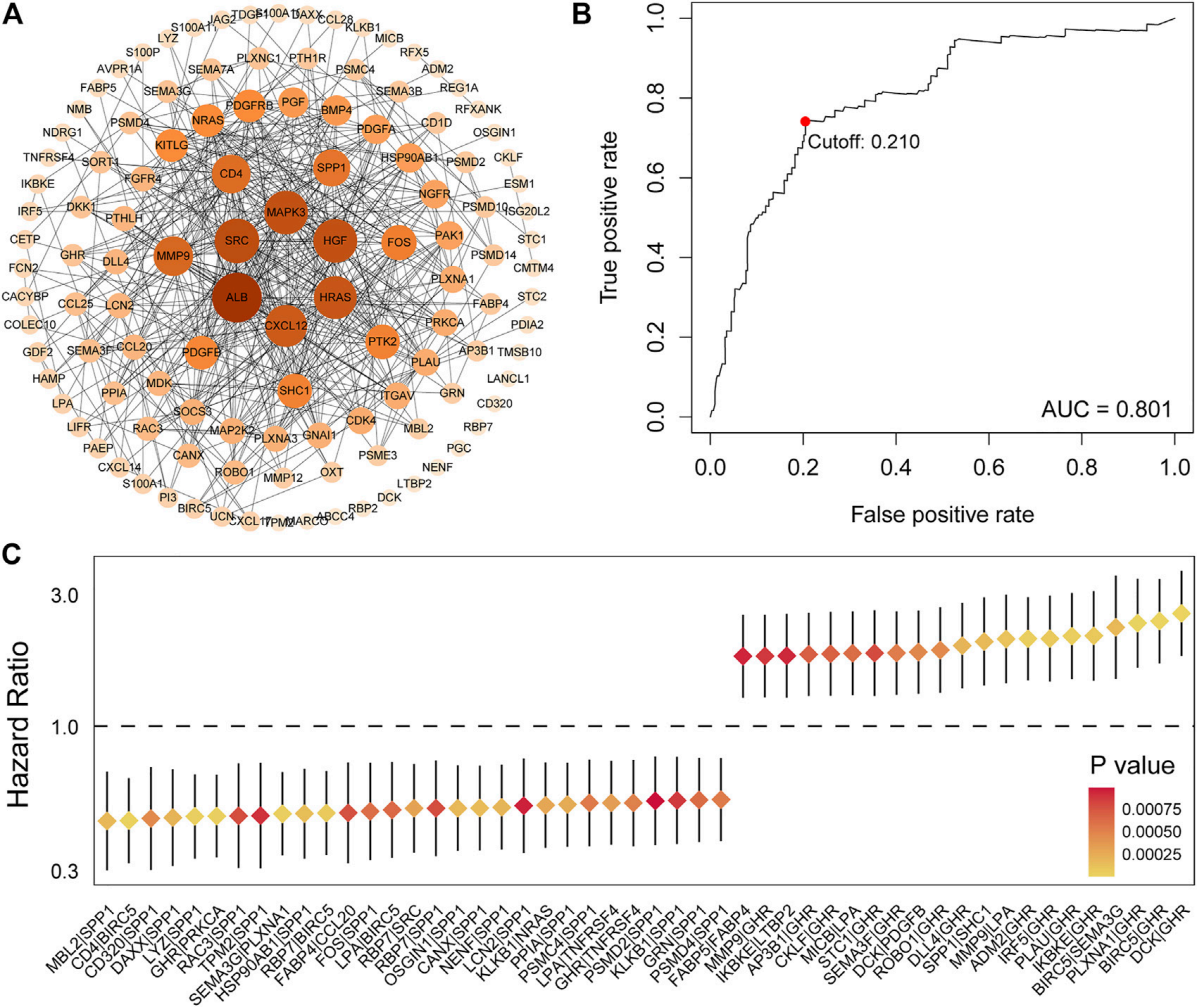
Identification of IRGs and determination of risk score. **(A)** 120 shared differential immunity related genes were displayed on the protein-protein interaction network. **(B)** Univariate Cox analysis obtained 50 IRGPs related to prognosis. **(C)** ROC curve of the risk score of the IRGPs signature in the TCGA cohort. The risk score was 0.210, which served as a threshold for classifying patients into the high and low-risk patients.

**FIGURE 2:**
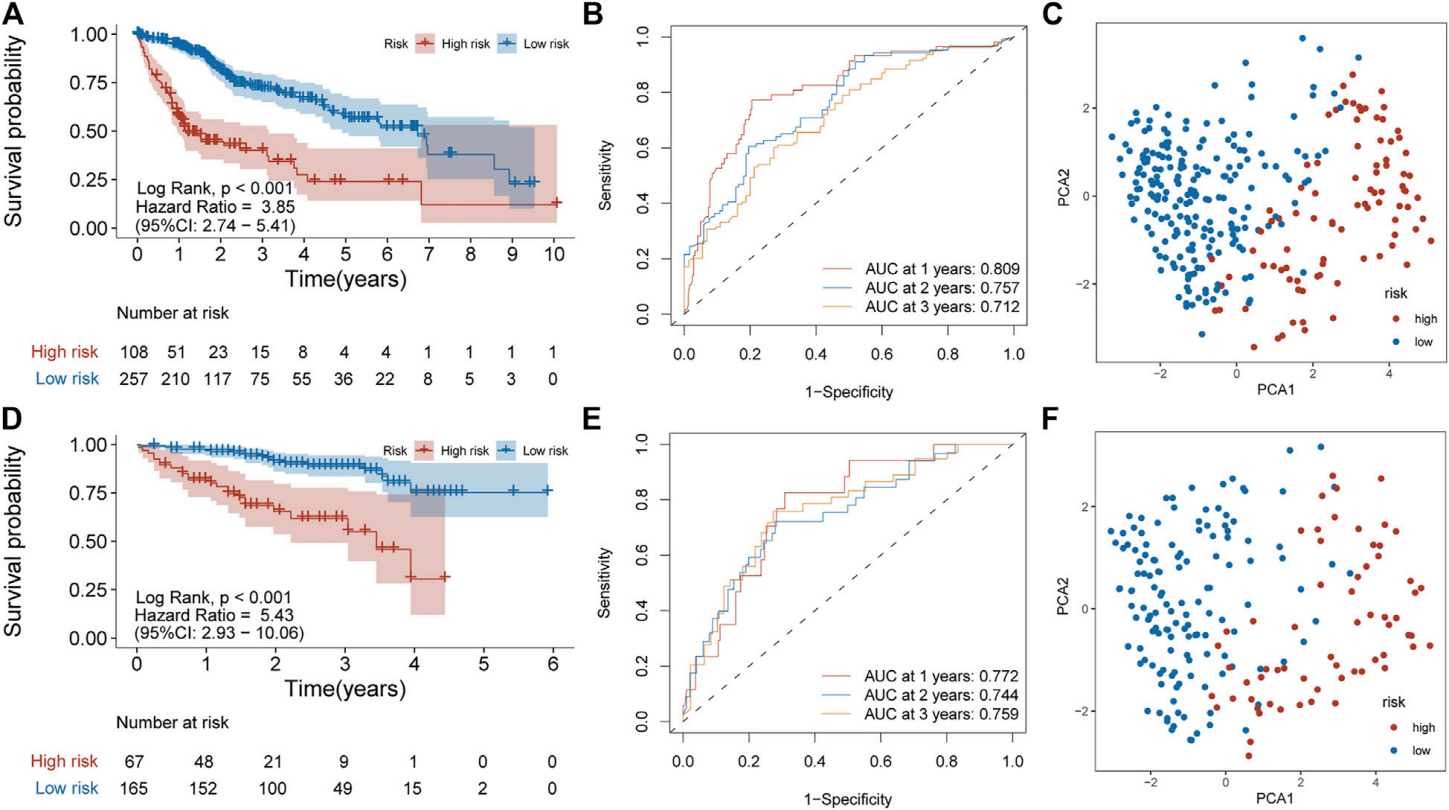
Prognostic analysis of the IRGPs signature in TCGA and ICGC cohorts. **(A)** Kaplan-Meier curve of overall survival in the high- and low-risk patients in the TCGA cohort. **(B)** ROC curve in TCGA cohort. **(C)** PCA plot of TCGA cohort. **(D)** Kaplan-Meier curve of overall survival in the high- and low-risk patients in the ICGC cohort. **(E)** ROC curve in ICGC cohort **(F)** PCA plot of ICGC cohort.

Principal component analysis (PCA) was first performed on the expression profiles of the two cohorts. The results showed that there were differences in the distribution of tumor tissue and normal tissue in the two data sets, which could be used for further analysis (Supplementary Figures S1A,C). 231 differential IRGs were identified in the TCGA cohort and 172 differential IRGs were identified in the ICGC cohort (Supplementary Figures S1B,D). We obtained 120 shared differential IRGs and displayed them in the protein-protein interaction network (Figure 1A). In order to obtain genes with high variability, we screened among 120 IRGs. The filter criterion was that the median absolute deviation of these genes in tumor samples is greater than 1. 90 IRGs were finally got for constructing 1,309 pairs of IRGPs. The correlation between these IRGPs and overall survival was evaluated in the TCGA cohort, and 50 IRGPs related to prognosis were screened out, with *p* < 0.001 as the cut-off criterion (Figure 1C). A signature composed of 17 IRGPs was constructed on the TCGA cohort through LASSO Cox proportional hazard regression. The IRGPs signature consists of 22 unique IRGs (Supplementary Table S1). Finally, the time-dependent ROC curve was used to determine the optimal cutoff value of 0.210 for the risk score, classified patients as high- or low-risk patients (Figure 1B). At this point, the sensitivity was 74.1% and the specificity was 75.5%. In the TCGA cohort, the high-risk patients were found to be significantly correlated with histologic grade, Child-Pugh grade, alpha-fetoprotein, vascular invasion, and TNM stage (Table 1). Overall survival in the TCGA cohort was worse in the high-risk patients than in the low-risk patients (Log-rank, *p* < 0.001) (Figure 2A). PCA analysis shows that the distribution patterns of patients in different risk patients are contrasting (Figure 2B). The ROC curve assessed the prognostic ability of the prognostic signature, and the area under the curve (AUC) reaches 0.809 at 1 year, 0.757 at 2 years, and 0.712 at 3 years (Figure 2C).

**TABLE 1:** Baseline characteristics of the patients in different risk groups.

Variables	TCGA cohort				ICGC cohort			
	High risk	low risk	χ2	P	High risk	low risk	χ2	P
Age (year)	—	—	0	1	—	—	150.09	<0.001
<60	50 (50.0%)	119 (46.3%)	—	—	5 (16.4%)	36 (23.6%)	—	—
≧60	58 (50.0%)	138 (53.7%)	—	—	58 (83.6%)	129 (76.4%)	—	—
Gender	—	—	0.1	0.75	—	—	5.13	0.02
female	37 (34.3%)	82 (31.9%)	—	—	25 (37.3%)	36 (21.8%)	—	—
male	71 (65.7%)	175 (68.1%)	—	—	42 (62.7%)	129 (78.2%)	—	—
Histologic grade	—	—	19.71	<0.001	—	—	—	—
G1 and G2	50 (46.3%)	180 (70.0%)	—	—	—	—	—	—
G3 and G4	57 (52.8%)	73 (28.4%)	—	—	—	—	—	—
unknow	1 (0.9%)	4 (1.6%)			—	—	—	—
Child–Pugh grade		—	6.49	0.04	—	—	—	—
A	53 (49.1%)	163 (63.4%)	—	—	—	—	—	—
B and C	8 (7.4%)	14 (5.4%)	—	—	—	—	—	—
unknow	47 (43.5%)	80 (31.1%)	—	—	—	—	—	—
Alpha fetoprotein	—	—	15.69	<0.001	—	—	—	—
<200 ng/ml	43 (39.8%)	158 (61.5%)	—	—	—	—	—	—
≧200 ng/ml	33 (30.6%)	42 (16.3%)	—	—	—	—	—	—
unknow	32 (29.6%)	57 (22.2%)	—	—	—	—	—	—
Vascular invasion	—	—	20.46	<0.001	—	—	—	—
YES	40 (37.0%)	66 (25.7%)	—	—	—	—	—	—
NO	42 (38.9%)	163 (63.4%)	—	—	—	—	—	—
unknow	26 (24.1%)	28 (10.9%)	—	—	—	—		—
TNM stage	—	—	13.57	<0.001	—	—	9.76	<0.001
I and II	65 (60.2%)	189 (73.5%)	—	—	30 (44.8%)	112 (67.9%)	—	—
III and IV	39 (36.1%)	48 (18.7%)	—	—	37 (55.2%)	53 (32.1%)	—	—
unknow	4 (3.7%)	20 (7.8%)	—	—	—	—	—	—

### Validation of the Feasibility of the IRGPs Signature to Predict Survival

To determine whether the signature has prognostic value, the signature was applied to the ICGC cohort as independent external verification. Patients in the ICGC cohort were divided into high or low-risk patients based on the above risk score. The high-risk patients in the ICGC cohort were also correlated with the TNM stage (Table 1). Also, the overall survival rate of the high-risk patients in the ICGC cohort is lower than in the low-risk patients (Log-rank, *p* < 0.001) (Figure 2D). PCA analysis showed that the distribution pattern of the two groups of patients was distinct (Figure 2E). In addition, the ROC curve showed 0.772 at 1 year, 0.744 at 2 years, and 0.759 at 3 years (Figure 2F). These results are similar to those obtained in the TCGA cohort.

### Validation of the IRGPs Signature as an Independent Prognostic Factor

**FIGURE 3:**
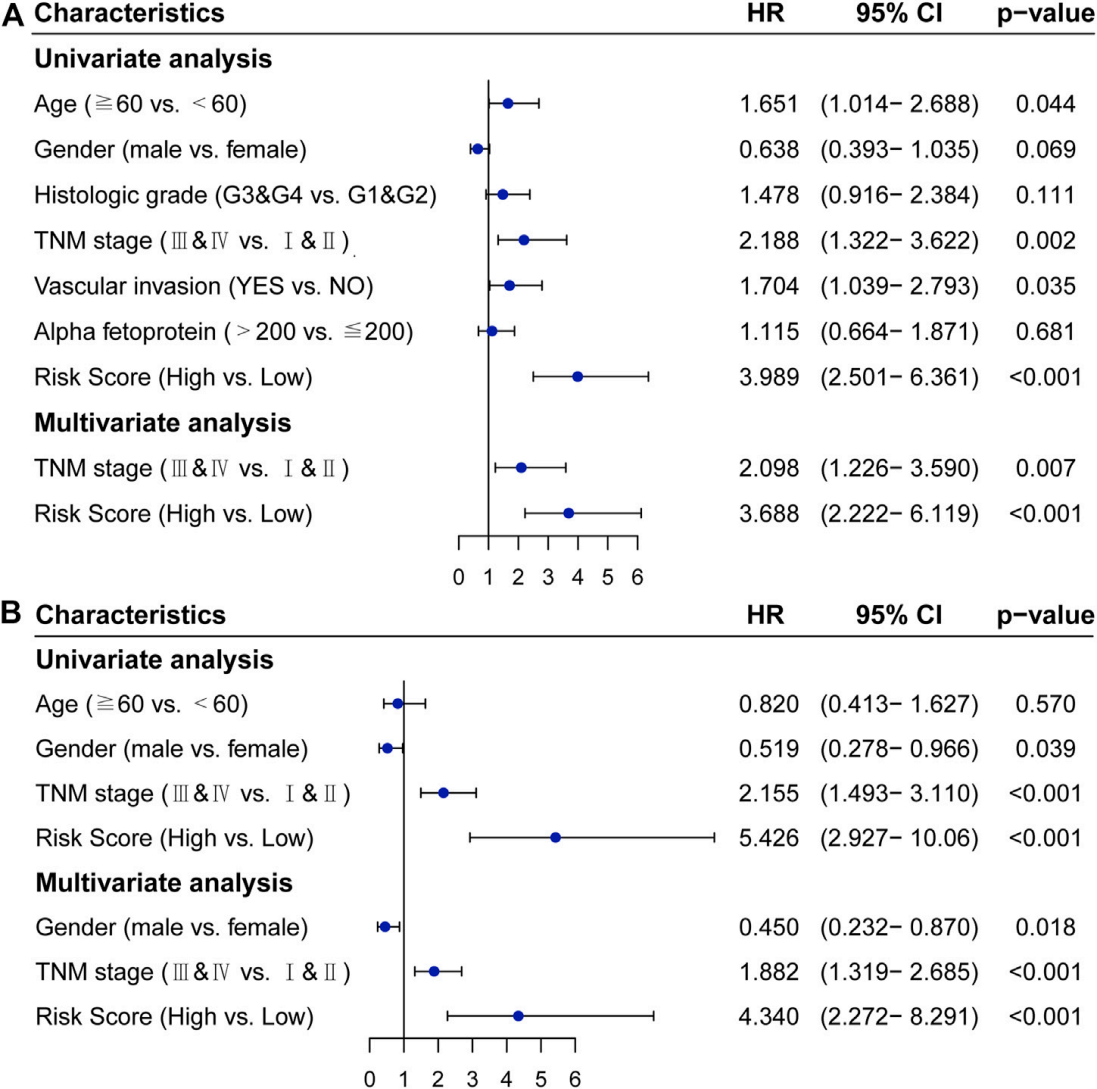
Cox proportional hazards regression model analysis of overall survival in patients with hepatocellular carcinoma. **(A)** TCGA cohort. **(B)** ICGC cohort. HR: hazard ratio, CI: confidence interval.

In the TCGA cohort, univariate COX analysis showed that TNM stage (*p* = 0.002), vascular invasion (*p* = 0.035), and the IRGPs signature (*p* < 0.001) were significantly correlated with the prognosis of HCC. After adjusting for clinical and pathological factors such as age, gender, histologic grade, TNM stage, vascular invasion, and alpha-fetoprotein, the TNM stage (HR, 2.098; 95%CI, 1.226,3.590; *p* = 0.007) and the IRGPs signature (HR, 3.688; 95%CI, 2.222,6.119; *p* < 0.001) were independent risk factors in the multivariate COX analysis (Figure 3A). Similarly, in the ICGC cohort, univariate COX analysis showed that TNM stage (*p* < 0.001) and the IRGPs signature (*p* < 0.001) were also significantly correlated with the prognosis of hepatocellular carcinoma. Multivariate COX analysis revealed that the TNM stage (HR, 1.882; 95%CI, 1.319,2.685; *p* < 0.001) and the IRGPs signature (HR, 4.340; 95% CI, 2.272, 8.291; *p* < 0.001) were also independent risk factors of overall survival (Figure 3B). In the TCGA and ICGC cohorts, high and low-risk patients have different distributions in the TNM stage (Supplementary Figures S2A,C). In addition, the IRGPs signature divided patients with early (I and II) and late (III and IV) HCC into different prognostic groups. Also, for patients with stage IandII disease, low-risk patients have a good prognosis in both the TCGA cohort (Log-rank, *p* < 0.001) and the ICGC cohort (Log-rank, *p* < 0.001) (Supplementary Figures S2B,D). Similarly, for patients with advanced stages III and IV, low-risk patients also have a good prognosis in the TCGA cohort (Log-rank, *p* < 0.001) and ICGC cohort (Log-rank, *p* = 0.01) (Supplementary Figures S2B,D). Overall, the IRGPs signature seems to be able to independently assess the overall survival of HCC.

### Functional Annotation and Immune Cell Infiltration Between High and Low-Risk Patients

**FIGURE 4:**
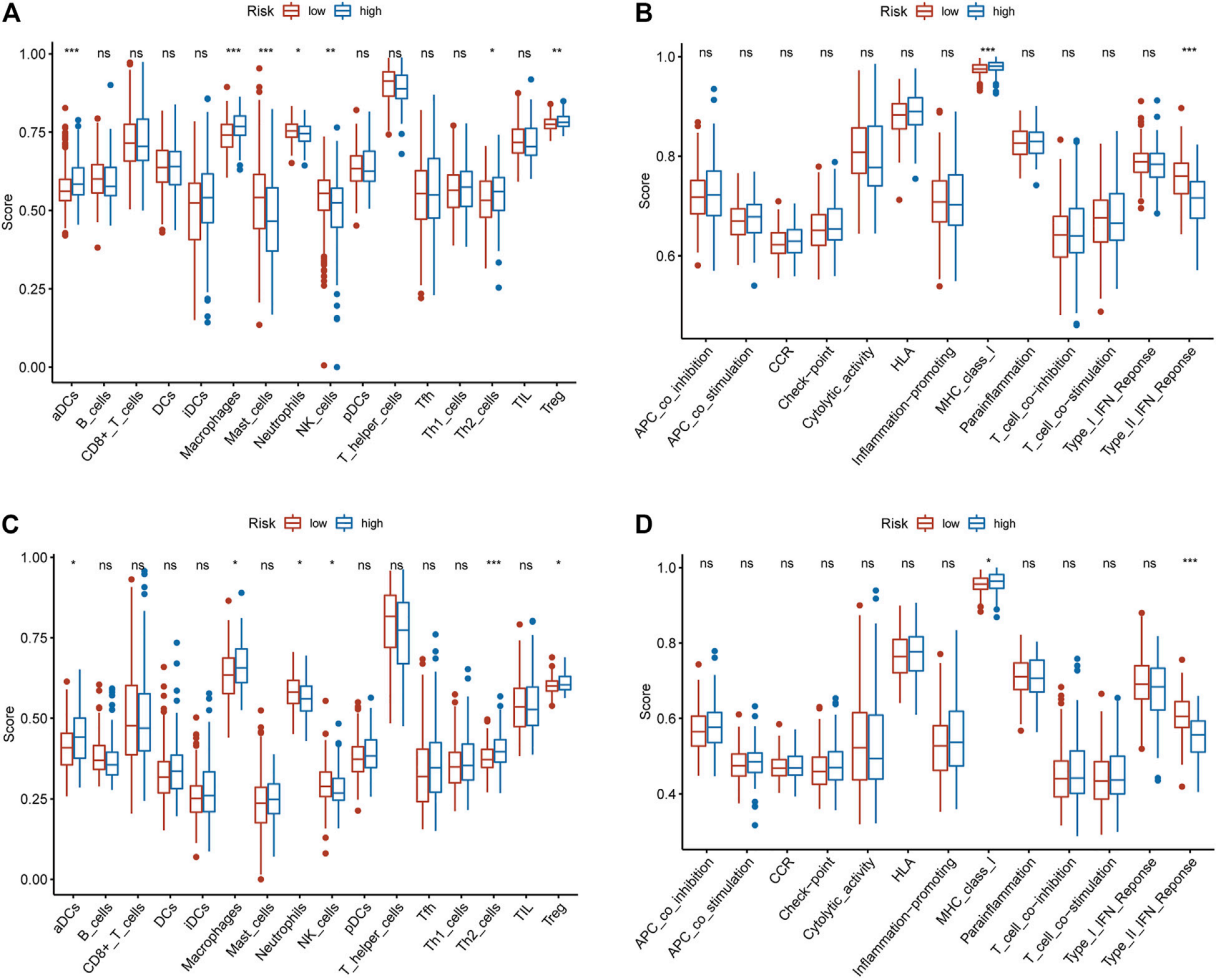
The relationship between the IRGPs signature and immune infiltrating cells and immune-related functions. **(A)** The relationship between the IRGPs signature and immune cells in the TCGA cohort. **(B)** The relationship between the IRGPs signature and immune-related functions in the TCGA cohort. **(C)** The relationship between the IRGPs signature and immune cells in the ICGC cohort. **(D)** The relationship between the IRGPs signature and immune-related functions in the ICGC cohort. The adjusted *p* value is ns, which is not significant. *p < 0.05; **p < 0.01; ***p < 0.001.

GSEA results showed that some immune-related biological processes are involved in high-risk patients (Supplementary Figure S3A), such as activation of innate immune response, antigen processing and presentation of peptide antigen via MHC class I, positive regulation of activated T cell proliferation, regulation of type I interferon mediated signalling pathway. Interestingly, some immune-related KEGG pathways are enriched in high-risk patients (Supplementary Figures S3B), such as FC epsilon RI signalling pathway, MAPK signalling pathway, mTOR signalling pathway, NOD like receptor signalling pathway. These results indicate that immune-related biological processes may play an indispensable role in the development of HCC. We further explored the status of immune cells and immune-related functions in high-risk and low-risk populations. In the TCGA cohort, the high-risk patients were positively correlated with tumor-infiltrating immune cells (aDCs, Macrophages, TH2 cells, Treg), while negatively correlated with Mast cells, Neutrophils, and NK cells (Figure 4A). In the ICGC cohort, except for Mast cells, which were not statistically significant, the results were consistent with the results in TCGA (Figure 4C). In the TCGA cohort, the high-risk patients were positively correlated with MHC class I and negatively correlated with Type II IFN Response (Figure 4B). In the ICGC cohort, the same result was obtained (Figure 4D).

### Reactivity of Immune Checkpoint Therapy and Sensitivity of Targeted Drugs

**FIGURE 5:**
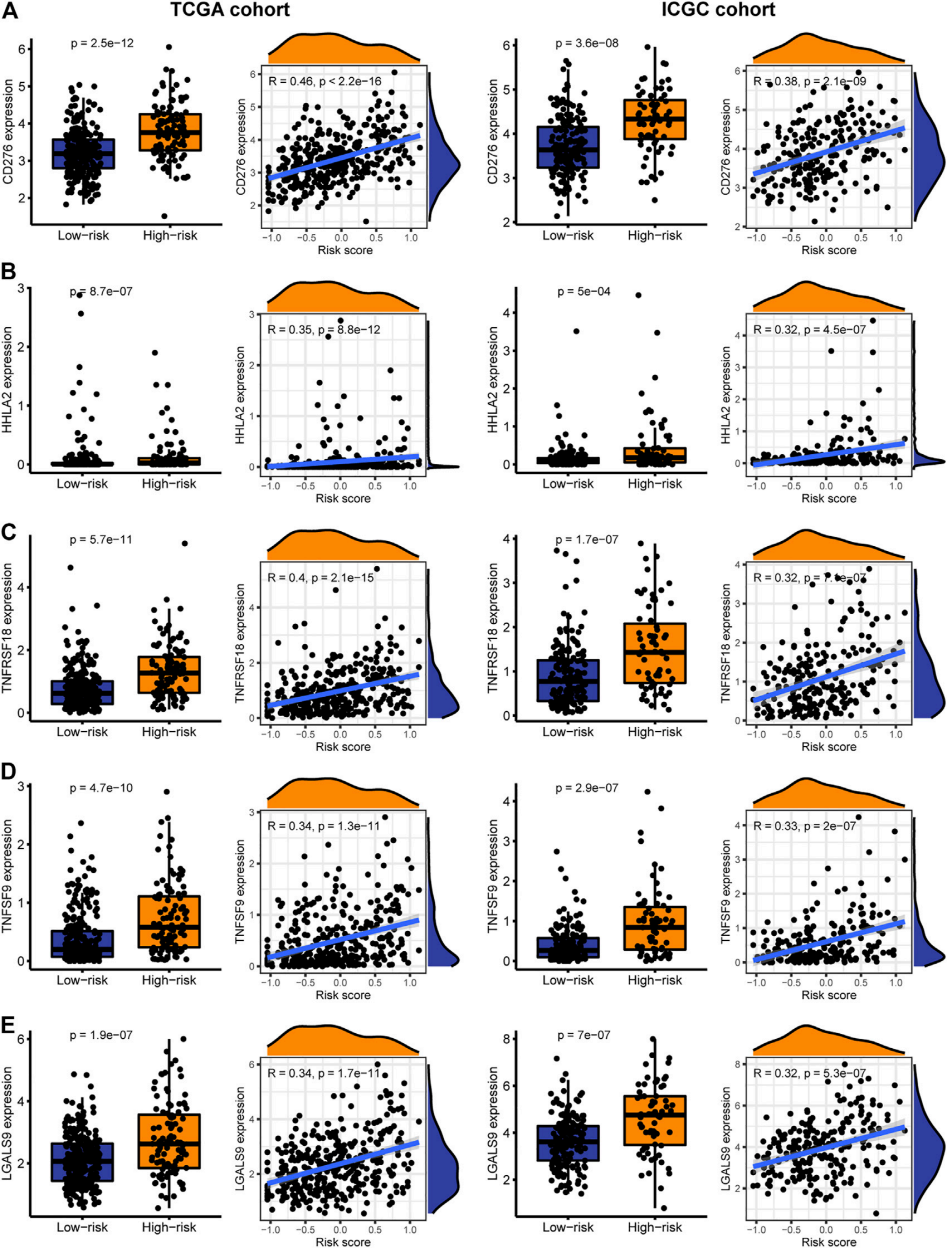
The expression and correlation between immune checkpoints in high and low-risk patients in the TARGET and ICGC cohorts. **(A)** CD276 **(B)** HHLA2 **(C)** TNFRSF18 **(D)** TNFSF9 **(E)** LGALS9.

**FIGURE 6:**
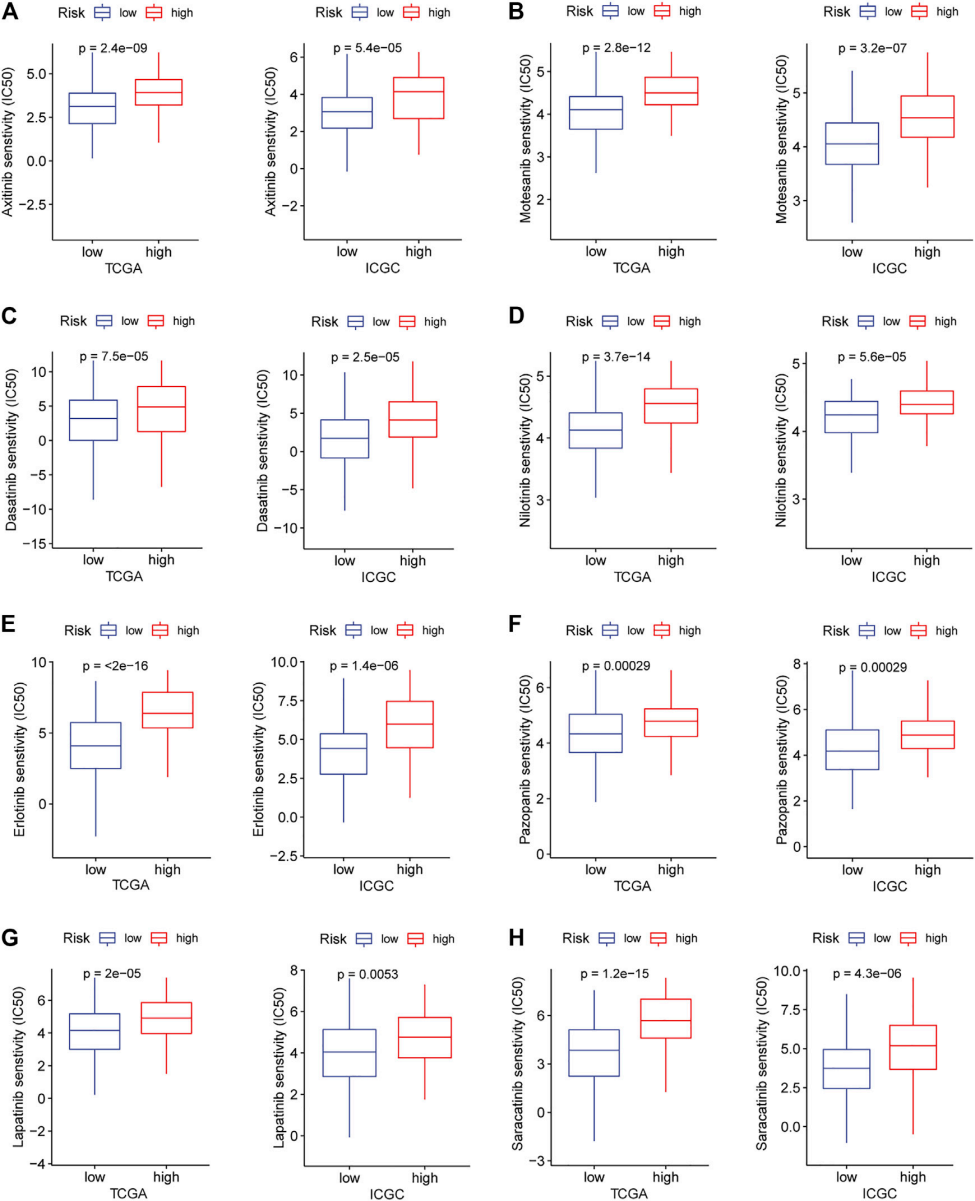
In the TARGET and ICGC cohorts, high and low-risk patients and targeted drug sensitivity. **(A)** Axitinib **(B)** Motesanib **(C)** Dasatinib **(D)** Nilotinib **(E)** Erlotinib **(F)** Pazopanib **(G)** Lapatinib **(H)** Saracatinib.

Immune checkpoint therapy has shown better results in the treatment of cancer, and it has made a major breakthrough in the field of HCC. Therefore, we investigated the expression of immune checkpoint markers in low and high-risk patients. In the TCGA and ICGC cohorts, immune checkpoint markers (CD276, HHLA2, TNFRSF18, TNFSF9, LGALS9) were expressed higher in high-risk patients, and there was a positive correlation between the IRGPs signature and these markers (|R| > 0.3, *p* < 0.05, Figures 5A–E). Tyrosine kinase inhibitors currently approved and in clinical trials have demonstrated efficacy in the treatment of hepatocellular carcinoma, which can be helpful in the treatment and management of patients with HCC. Hence, we evaluated the IC50 of each sample and observed that the IC50 of the eight small molecule tyrosine kinase inhibitors was significantly different between the two groups. The results showed that Axitinib, Motesanib, Dasatinib, Nilotinib, Erlotinib, Pazopanib, Lapatinib, and Saracatinib are more sensitive to low-risk patients (*p* < 0.001, Figures 6A–H). This may provide an accurate strategy for the treatment of HCC patients.

## Discussion

Immune infiltration plays an important role in cancer progression. With the exploration in the field of immunotherapy, it is of great help in the treatment of tumor. There have been studies that have provided strong evidence for the treatment and diagnosis of some diseases through bioinformatics analysis (Kumar et al., 2019; Udhaya Kumar et al., 2020). Due to the high heterogeneity of HCC, some patients fail to achieve the expected curative effect on immunotherapy. Therefore, it is extremely crucial to determine the sensitivity of different patient subsets to treatment response. Individualized treatment for different patient subgroups will help to enhance the prognosis of patients (Gan et al., 2020; Zhou et al., 2020; Kaur et al., 2021). Novel signature related to tumor immune infiltration may be a sword for identifying new molecular targets and improving patient prognosis.

In this study, we developed a prognostic signature based on 17 IRGPs in HCC and validated them in two independent data sets on different platforms. Our prognostic immune signature can further divide clinically defined patients [for example, early stage (I and II) and late stage (III and IV)] into subgroups with different survival outcomes. Univariate COX analysis showed that TNM stage and the IRGPs signature were significantly correlated with the prognosis of HCC. By multivariate COX analysis, the TNM stage and the prognostic signature can be used as independent prognostic factors. Therefore, our prognostic signature can be used as a personalized prognosis and diagnosis and treatment of HCC patients and can be easily translated into clinical practice.

The IRGPs signature we constructed included 22 IRGs, and the results of GSEA indicated that some immune-related biological processes and signal pathways were enriched in high-risk patients. Such as, activation of innate immune response, MAPK signalling pathway and mTOR signalling pathway, etc. Innate immunity is a part of the HCC tumor microenvironment, which can suppress and promote cancer. For example, dendritic cells, neutrophils, and macrophages can promote the occurrence of HCC, while natural killer cells and natural killer T cells can inhibit the development of HCC (Ruf et al., 2021). The activation of MAPK signalling pathway is closely related to the development of tumors, and it is activated in about 50% of patients with early HCC and almost all patients with advanced HCC (Neuzillet et al., 2014). Excessive activation of mTOR promotes the development of tumors, and affects the immune regulation involved in the differentiation of immune cells, and plays an important role in tumor metabolism (Zou et al., 2020). There may be a link between aberrant activation of these pathways for the low overall survival of high-risk patients. We further studied the relationship between tumor immune infiltration and the IRGPs signature. In the high-risk patients of the TCGA and ICGC cohorts, aDCs, Macrophages, TH2 cells, and Treg infiltration increased. Studies have shown that the increase of regulatory DCs can promote the increase of Treg in liver cancer (Cheng et al., 2016). Treg and Macrophages, which are less infiltration in HCC, have a good prognosis (Zhu et al., 2008; Zhou et al., 2016). The increase of TH2 cells is associated with poor prognosis of HCC (Zhu et al., 2008; Zhou et al., 2010). In this study, in the TCGA and ICGC cohorts, the infiltration of Neutrophils and NK cells in high-risk patients was reduced, while the prognosis of the high-risk patients was worse than that of the low-risk patients. Studies have pointed out that neutrophils can inhibit the development of cancer (Souto et al., 2011). NK cells infiltrate less in advanced HCC and are associated with poor prognosis (Wu et al., 2013). These results are consistent with our research results. In the high-risk patients in the TCGA and ICGC cohorts, the type II IFN reactivity decreased, while the MHC class I activity increased. Type II IFN is mainly produced by activated NK cells (Perussia, 1991), and it plays an important role in regulating the tumor immune environment. In our study, the NK cell invasion and type II IFN reactivity in the high-risk patients were reduced, and the prognosis of the high-risk patients was poor, which was consistent with the above study results. The level of MHC class I is elevated in patients with advanced HCC, and it may negatively regulate innate immunity and adaptive immunity to cause tumor escape mechanisms to occur (Jinushi et al., 2005). According to the above findings, dysregulation of the immune microenvironment may account for the survival differences between the IRGPs signature subgroups.

Among the immune checkpoint markers, CD276, HHLA2, TNFRSF18, TNFSF9, and LGALS9 were highly expressed in high-risk patients, indicating differences in responsiveness to these immune checkpoint treatments among patients grouped by this signature. CD276 plays an important role in innate immunity and T cell-mediated adaptive immunity, which is highly expressed in HCC and other cancers and is associated with poor patient prognosis. And it has great potential for immunotherapy (Picarda et al., 2016). HHLA2 can inhibit the function of CD4 and CD8 T cells, and blocking HHLA2 can enhance the proliferation and activation of T cells, which is helpful for cancer immunotherapy (Zhao et al., 2013; Rieder et al., 2021). TNFRSF18, also known as glucocorticoid-induced TNFR-related protein (GITR), is a costimulatory receptor in malignant tumors. Agonistic targeting of GITR can enhance the anti-tumor response of TIL derived from HCC patients (van Beek et al., 2019). TNFSF9, also known as 4-1BBL, is expressed on active T cells and antigen cells. 4-1BBL targeted immunotherapy has shown anti-tumor effects in the treatment of HCC (Xiao et al., 2007; Li et al., 2011). The high expression of LGALS9 is associated with the poor prognosis of many human cancers. LGALS9 preferentially kills T cells rather than cancer cells may assist cancer immune escape (Yang et al., 2021).

Several tyrosine kinase inhibitors have been used as first or second-line agents in the treatment of liver cancer, but one challenge is the lack of reliable biomarkers to identify patients who benefit from these treatments (Qin et al., 2019). Interestingly, we found that low-risk patients are more sensitive to Axitinib, Motesanib, Dasatinib, Nilotinib, Erlotinib, Pazopanib, Lapatinib, and Saracatinib. These findings provide effective treatment strategies for patients stratified by the IRGPs signature.

Although we used two independent data sets to rigorously verify the signature we proposed, our research has certain limitations. First of all, our study only carried out retrospective research and lacked prospective research. Second, our study only includes immune-related genes, and important prognostic genes in HCC may have been deleted. Finally, the relationship between the IRGPs signature and immune infiltration will be verified in follow-up studies.

In conclusion, the proposed IRGPs signature can accurately predict the prognosis of HCC patients and guide clinicians to make specific treatment decisions. It also provides the relationship between this signature and the responsiveness of immune checkpoints and targeted drugs. These results will be beneficial to the effective treatment of HCC patients. Prospective studies are required to further verify its accuracy.

## Data Availability

Publicly available datasets were analyzed in this study. This data can be found here: https://portal.gdc.cancer.gov/; https://daco.icgc.org/; https://www.immport.org/home.

## Abbreviations

AUC, area under curve; GO, gene ontology; GSEA, gene set enrichment analysis; HCC, hepatocellular carcinoma; HR, hazard ratio; ICGC, international cancer genome consortium; IRG(s), immune-related gene(s); IRGP(s), immune-related gene pair(s); KEGG, kyoto encyclopedia of genes and genomes; LASSO, least absolute shrinkage and selection operator; PCA, principal component analysis; ROC, receiver operating characteristic; TCGA, the cancer genome atlas.
