# Supplementary material for: Development and Verification of an Immune-Related Gene Pairs Prognostic Signature in Hepatocellular Carcinoma
Source: Front Mol Biosci. 2021 Oct 1;8:715728. doi: 10.3389/fmolb.2021.715728 (PMC8517445; doi:10.3389/fmolb.2021.715728)
Supplement: Supplementary file 1 [file DataSheet1.docx]

Development and Verification of an Immune-related Gene Pairs Prognostic Signature in Hepatocellular Carcinoma

Supplementary Material

# Supplementary Figures and Tables

## Supplementary Figures


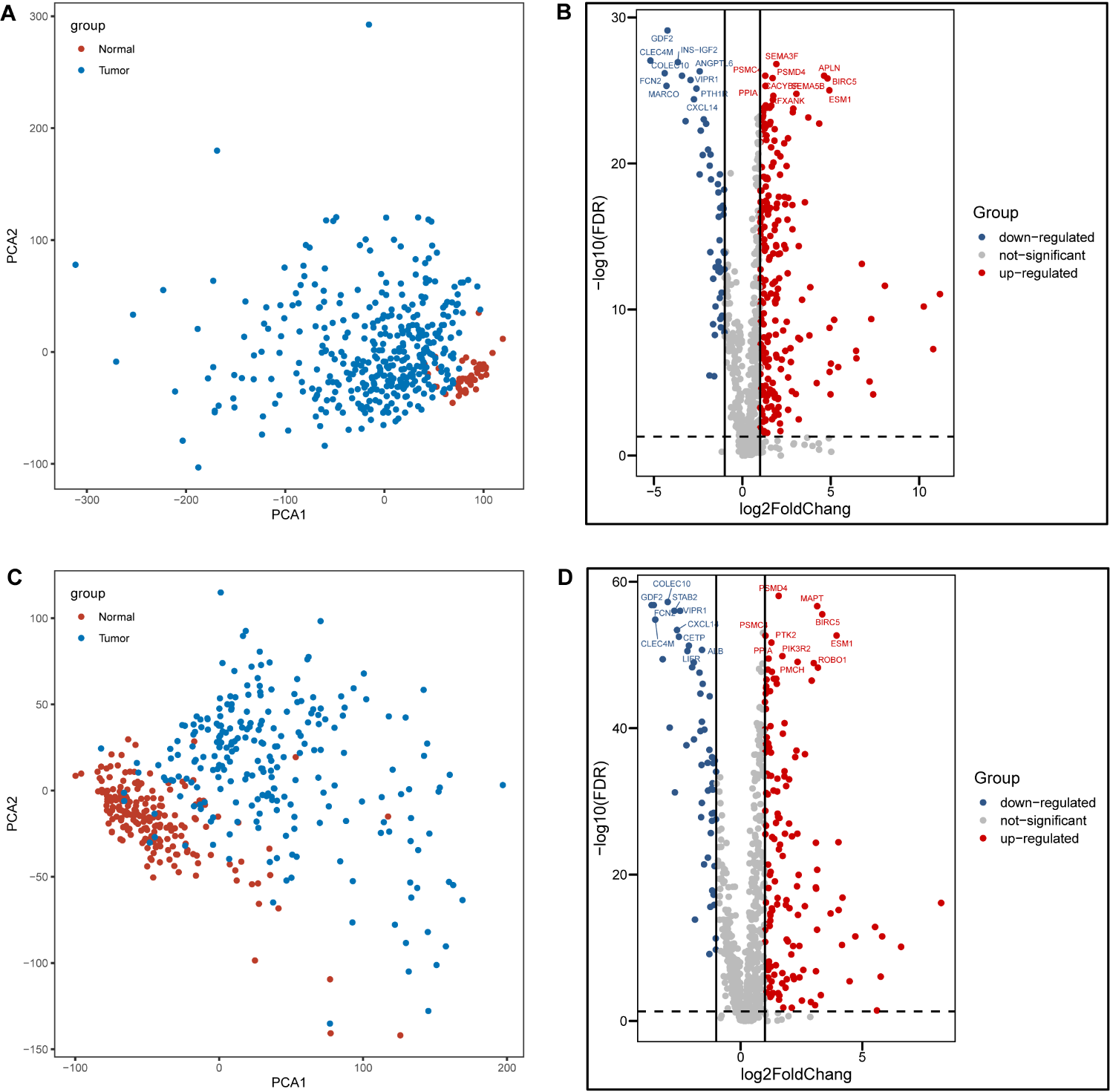


**Figure 1.** A. PCA plot of TCGA cohort. B. Volcano map of differential immunity-related genes in the TCGA cohort. C. PCA plot of ICGC cohort. D. Volcano map of differential immunity-related genes in the ICGC cohort.


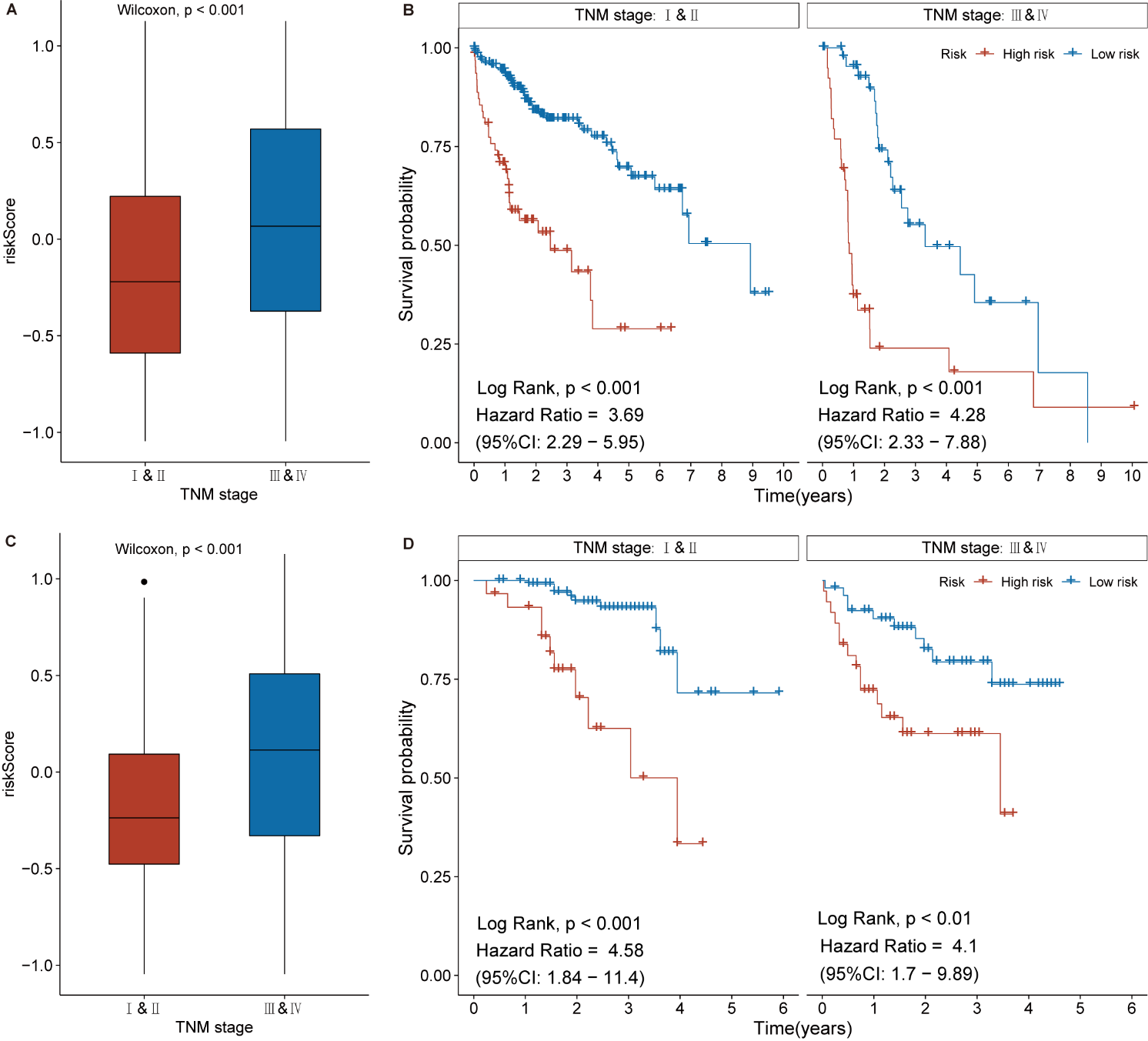


**Figure 2.** The relationship between TNM stage and the IRGPs signature. A. The relationship between the IRGPs signature and TNM stage in the TCGA cohort. B. Kaplan-Meier curve of overall survival for early- and late-stage patients with different IRGPs signature in the TCGA cohort. C. The relationship between the IRGPs signature and TNM stage in the ICGC cohort. D. Kaplan-Meier curve of overall survival for early- and late-stage patients with different the IRGPs signature in the ICGC cohort.


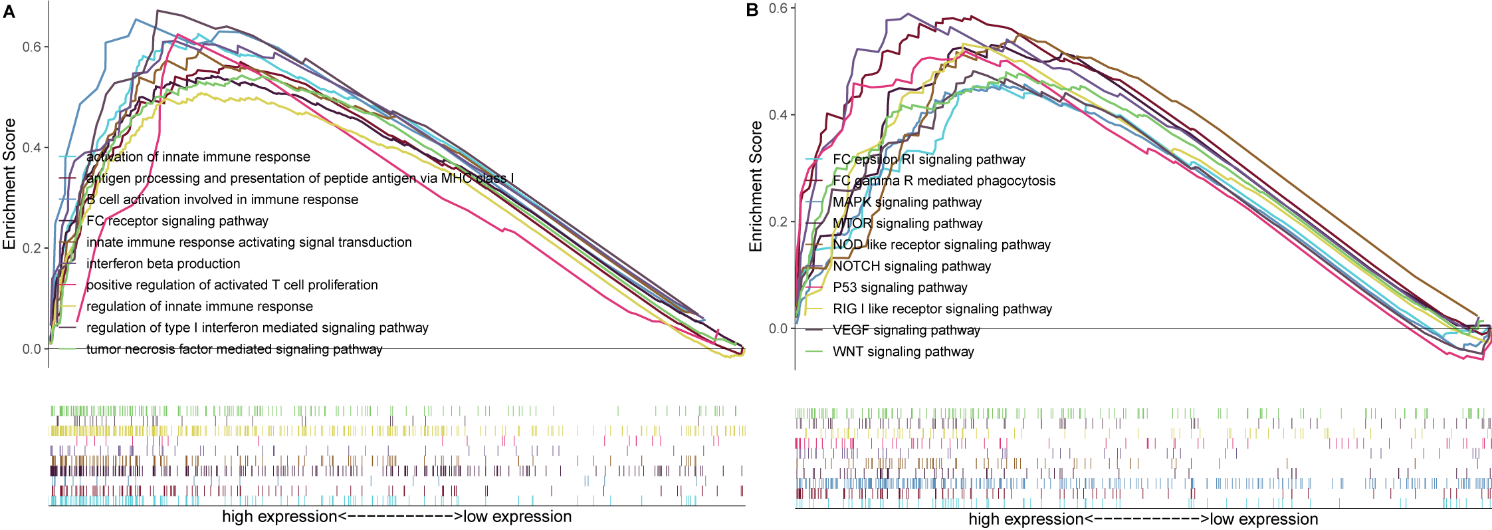


**Figure 3.** The corresponding biological functions of high-risk populations. A. GSEA analysis of biological processes in high-risk populations. B. GSEA analysis of KEGG pathway in high-risk populations.

## Supplementary Tables

**Table 1.** Model information about IRGPs signature

| IRG 1 | Full name | Immune processes | IRG 2 | Full name | Immune processes | Coefficient |
| --- | --- | --- | --- | --- | --- | --- |
| CD4 | CD4 molecule | Antigen Processing and Presentation | BIRC5 | baculoviral IAP repeat-containing 5 | Antimicrobials | -0.24706063 |
| HSP90AB1 | heat shock protein 90kDa alpha (cytosolic), class B member 1 | Antigen Processing and Presentation | SPP1 | secreted phosphoprotein 1 | Cytokines | -0.160843379 |
| MICB | MHC class I polypeptide-related sequence B | NaturalKiller Cell Cytotoxicity | LPA | lipoprotein, Lp(a) | Antimicrobials | 0.073197944 |
| LCN2 | lipocalin 2 | Antimicrobials | SPP1 | secreted phosphoprotein 1 | Cytokines | -0.238687948 |
| LPA | lipoprotein, Lp(a) | Antimicrobials | TNFRSF4 | tumor necrosis factor receptor superfamily, member 4 | Cytokine Receptors | -0.035200939 |
| FABP4 | fatty acid binding protein 4, adipocyte | Antimicrobials | CCL20 | chemokine (C-C motif) ligand 20 | Antimicrobials | -0.204937187 |
| RBP7 | retinol binding protein 7, cellular | Antimicrobials | SRC | v-src sarcoma (Schmidt-Ruppin A-2) viral oncogene homolog (avian) | Antimicrobials | -0.007290982 |
| LYZ | lysozyme (renal amyloidosis) | Antimicrobials | SPP1 | secreted phosphoprotein 1 | Cytokines | -0.143825942 |
| DLL4 | delta-like 4 (Drosophila) | Antimicrobials | GHR | growth hormone releasing hormone | Cytokines | 0.154232185 |
| DCK | deoxycytidine kinase | Antimicrobials | PDGFB | platelet-derived growth factor beta polypeptide (simian sarcoma viral (v-sis) oncogene homolog) | Cytokines | 0.210699846 |
| DCK | deoxycytidine kinase | Antimicrobials | GHR | growth hormone releasing hormone | Cytokines | 0.287759203 |
| BIRC5 | baculoviral IAP repeat-containing 5 | Antimicrobials | SEMA3G | sema domain, immunoglobulin domain (Ig), short basic domain, secreted, (semaphorin) 3G | Chemokines | 0.153367061 |
| BIRC5 | baculoviral IAP repeat-containing 5 | Antimicrobials | GHR | growth hormone releasing hormone | Cytokines | 0.047929659 |
| PLXNA1 | plexin A1 | Chemokine Receptors | GHR | growth hormone releasing hormone | Cytokines | 0.029255843 |
| ROBO1 | roundabout, axon guidance receptor, homolog 1 (Drosophila) | Chemokine Receptors | GHR | growth hormone releasing hormone | Cytokines | 0.060570303 |
| ADM2 | adrenomedullin 2 | Cytokines | GHR | growth hormone releasing hormone | Cytokines | 0.11141906 |
| GHR | growth hormone releasing hormone | Cytokines | PRKCA | protein kinase C, alpha | NaturalKiller Cell Cytotoxicity | -0.007772174 |
